# Impact of exercise on drug cravings: mediating role of cardiorespiratory fitness and inhibitory control

**DOI:** 10.3389/fpsyg.2025.1540648

**Published:** 2025-04-09

**Authors:** Boya Li, Yuehui Zhou, Youling Qian, Juhua Wu

**Affiliations:** ^1^School of Sport Science, Qufu Normal University, Qufu, Shandong, China; ^2^School of Physical Education, Hubei Minzu University, Hubei, Enshi, China; ^3^School of Sport, Guangxi University of Science and Technology, Liuzhou, Guangxi, China

**Keywords:** cardiorespiratory fitness, physical exercise, drug cravings, psychological craving, inhibition, rehabilitation efforts

## Abstract

**Background:**

Contemporary research has consistently demonstrated a link between physical exercise, inhibition, and drug cravings, with several hypotheses proposed to explain how exercise enhances inhibition. However, few studies have explored the mechanisms underlying this effect. This study investigates the pivotal role of cardiorespiratory fitness in mediating the impact of physical exercise on inhibition and drug cravings.

**Methods:**

In this study, the researchers selected participants who had completed physical detoxification in China’s compulsory isolation drug rehabilitation centers. Moreover, we conducted surveys and assessed cardiorespiratory fitness using tools such as the Physical Exercise Rating Scale, Inhibition Scale, Drug Craving Scale, and Queen College Step Test. Additionally, we employed exploratory and confirmatory factor analyses, correlation analysis, regression analysis, and structural equation model (SEM) to analyze the study’s data.

**Results and findings:**

The findings of this study reveal that physical exercise significantly reduces drug craving by improving cardiorespiratory fitness and enhancing inhibition. Furthermore, it showed that men had better physical exercise levels, cardiorespiratory fitness, and inhibition compared to women, while women exhibited higher psychological cravings. Besides, the study also highlighted that longer years of drug use were associated with lower physical exercise, reduced cardiorespiratory fitness, and weaker inhibition, leading to higher drug cravings. In addition, cardiorespiratory fitness and inhibition acted as mediators between physical exercise and drug craving, with the combination of both factors serving as a chain mediator in reducing cravings. These findings suggest that physical exercise, particularly through improving cardiorespiratory fitness, plays a key role in mitigating drug cravings and supporting rehabilitation efforts for drug dependence.

## Introduction

The World Drug Report 2024 highlights the escalating impact of the global drug problem, fueled by the emergence of synthetic opioids and increased drug supply and demand (Nations, 2024). Despite this trend, China, has achieved notable success in reducing drug abuse, with the number of active drug addicts declining for six consecutive years and the rate of sustained abstinence rising steadily from 2013 to 2024 (Wang et al., 2023; Committee, C. n. n. c, 2024). This progress is largely credited to China’s unified “four areas and five centers” drug treatment model, which integrates detoxification, education, rehabilitation, and reintegration with specialized centers for medical care, psychological support, and skill development (P.R.C, M. O. J, 2024). Notably, the promotion of exercise interventions by China’s judicial administration since 2018 has played a crucial role in these achievements (Wang et al., 2023). Exercise, recognized as an alternative therapy for drug dependence since the early 21st century (Weber et al., 2004), has been shown to significantly reduce psychological cravings for drugs (Lynch et al., 2013; Weinstock et al., 2016; Zhang and Yuan, 2019; Ding et al., 2024). However, the mechanisms through which exercise mitigates cravings remain poorly understood.

Long-term drug abuse damages brain regions like the hippocampus and amygdala, which are critical for learning and memory while reinforcing the brain’s reward center and impairing inhibitory control. During withdrawal, weakened cognitive control allows drug-related stimuli to dominate emotions, behavior, and attention, triggering impulsive and compulsive drug-seeking behaviors (Baler and Volkow, 2006; Goldstein and Volkow, 2011). This highlights reduced inhibition as a key driver of drug cravings. Research indicates that physical exercise can enhance inhibitory control and reduce cravings, particularly in individuals recovering from methamphetamine and heroin dependence (Dongshi et al., 2015; Dongshi et al., 2020). Moderate-intensity exercise appears especially beneficial (Wang et al., 2016; Wang et al., 2019; Jin et al., 2024), with evidence suggesting that inhibition partially mediates the relationship between exercise and reduced cravings (Wang et al., 2019; Zhang et al., 2021). However, few studies have examined the underlying mechanisms by which exercise improves inhibitory capacity in drug addicts, limiting the potential for developing targeted exercise-based interventions for preventing and treating drug cravings.

Numerous studies reveal that individuals dependent on drugs, alcohol, or nicotine often exhibit impaired cardiorespiratory fitness. Physical exercise has been shown to reverse this damage, reduce cravings, and suggest that improved cardiorespiratory fitness may decrease drug cravings (Brown et al., 2010; Fischer et al., 2012; Morris et al., 2018; Zhou et al., 2024), this holds significant importance for optimizing treatment strategies, as it offers a non-pharmacological therapeutic approach that enhances the targeting of interventions. In the late 1990s, Kramer proposed the “cardiorespiratory fitness hypothesis” in Nature, suggesting that exercise-induced improvements in cardiorespiratory fitness drive cognitive benefits (Kramer et al., 1999). Subsequent research confirmed that physical exercise leads to neurophysiologic adaptations, including increased blood flow to frontal and parietal regions, cerebral angiogenesis, upregulation of neurotrophic factors, and neurogenesis. These changes enhance brain health and executive functions, particularly inhibition (Garber et al., 2011; Billinger et al., 2017; Zhang et al., 2023).

**Figure 1:**
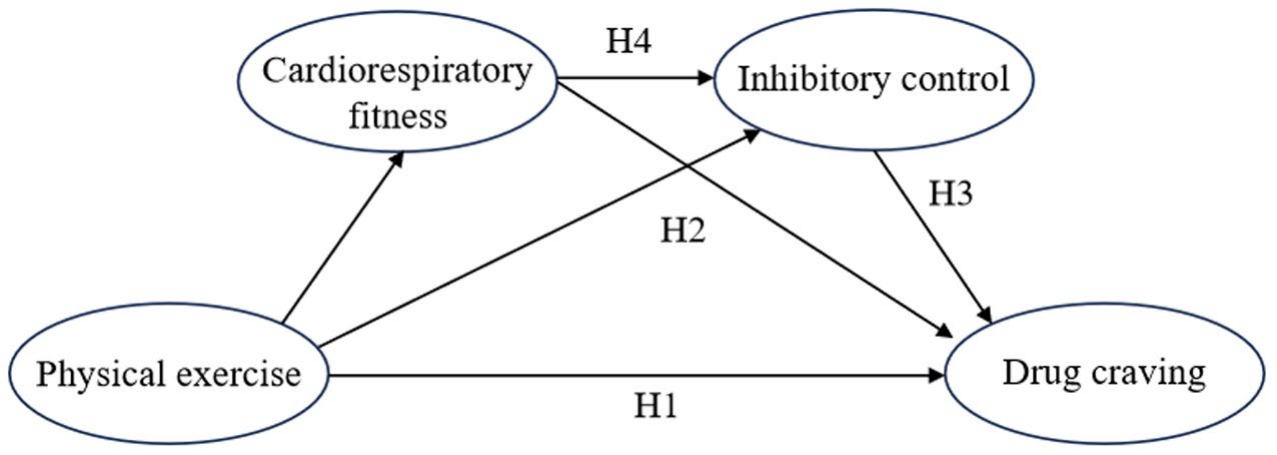
Hypothesis model diagram of chain mediating effect on physical exercise and drug craving.

Congruently, physical exercise reduces cravings in drug addicts, with inhibition acting as a mediator, serving as the core component of the mechanism. This is crucial for understanding the neural mechanisms underlying drug dependence and facilitates the development of more effective interventions. Exercise also uniquely improves cardiorespiratory fitness, further reducing cravings. Notably, cardiorespiratory fitness and inhibition are interconnected and benefit selectively from physical exercise. Exploring the core value of this mediating mechanism lies in its dual capacity to optimize existing treatment strategies, by enhancing cardiopulmonary health and inhibitory functions to mitigate drug cravings, and also improving the precision of interventions, enabling tailored therapeutic approaches that address individuals’ specific needs. While existing research has validated the relationship among above variables (Wang et al., 2019; Zhang et al., 2020), few studies have investigated the interconnected roles including cardiorespiratory fitness in a path model. Addressing this gap, the current study hypothesizes: (H1) Physical exercise is negatively correlated with drug cravings. (H2) Cardiorespiratory fitness mediates the relationship between physical exercise and drug cravings. (H3) Inhibitory control mediates this relationship, and (H4) Cardiorespiratory fitness and inhibitory control jointly play a chain-mediating role between physical exercise and drug cravings (see Figure 1).

## Subjects and methods

### Subjects

The researchers recruited participants with a history of drug abuse from three compulsory rehabilitation centers in Northwest and East China during their rehabilitation consolidation period. The inclusion criteria are as follows: (1) Currently in a state of incarceration with over 3 months of rehabilitation; (2) Meet DSM-IV diagnostic criteria for drug dependence through structured interview assessment; (3) No history of loss of consciousness due to head injury; (4) No personal or immediate family history of psychosis; (5) Cognitive assessment score of 5 points or higher; (6) Cleared by Physical Activity Readiness Questionnaire (PAR-Q) without physical disabilities or medical conditions preventing participation in moderate-intensity aerobic exercise.

Due to COVID-19 restrictions and strict management protocols, supervisors at the centers distributed and collected all questionnaires and conducted cardiorespiratory fitness tests. As these drug rehabilitation institutions serve as pilot units for exercise-based drug rehabilitation in China, and both questionnaire filling and cardiopulmonary fitness tests constitute parts of routine health education activities, the participants cooperated actively. Of the 360 questionnaires distributed, 346 were returned, and 305 valid samples were included after excluding 41 invalid responses, yielding an effective recovery rate of 84.72%. As shown in Table 1, among the participants, 272 (89.18%) were male, and 33 (10.82%) were female, with ages ranging from 19 to 61 years (mean age 37.26 ± 8.46). Most participants (67.87%) had an education level of junior high school or below. Regarding drug types, 35.41% used traditional drugs, while 64.59% used new synthetic drugs. On average, participants had a drug use history of 7.22 ± 4.42 years, with 44.26% reporting at least one relapse. All participants were informed of the study’s purpose and procedures and provided consent. The Biomedical Ethics Committee of Qufu Normal University approved the study protocol (No. 2021031).

**Table 1:** Basic characteristics of subjects.

Demographic variables		Mean ± SD/n (%)	Drug use/Exercise-related data		Mean ± SD/n (%)
Age (years)	From 19 to 61	37.26 ± 8.46	Drug types	Traditional drug	108 (35.41%)
Height (m)	From 1.55 to 1.88	1.74 ± 0.06		New drug	197 (64.59%)
Weight (kg)	From 51 to 111	78.12 ± 11.05	Relapse	0	170 (55.74%)
Sex	Male	272 (89.18%)		1	101 (33.11)
	Female	33 (10.82%)		2	22 (7.21%)
Education	Primary school or below	73 (23.93%)		≥3	12 (3.93%)
	Junior	134 (43.93%)	Drug use years	From 1 to 20	7.22 ± 4.42
	Senior	68 (22.30%)	Amount of exercise	From 1 to 64	15.41 ± 18.05
	College or above	30 (9.84%)	VO_2_max	From 25 to 39	32.93 ± 5.16
Career	Unemployed	67 (21.97%)	Intensity	Low	98 (32.13%)
	Self-employed	179 (58.69%)		Lower	78 (25.57%)
	Staff	37 (12.13%)		Moderate	54 (17.70%)
	Manual workers	22 (7.21%)		High	75 (24.59%)
			Time	21–30 min	143 (46.89%)
				31–40 min	101 (33.11%)
				41-50 min	37 (12.13%)
				≥51 min	24 (7.87%)
			Frequency	≤1 per wk	124 (40.66%)
				2–3 per wk	55 (18.03%)
				4 per wk	58 (19.02%)
				5 per wk	68 (22.30%)

## Research methods

### Measures

The study employed a structured questionnaire covering four main areas. The first section collected basic demographic and drug use information, including gender, age, drug types, and relapse history. The remaining three sections comprised validated scales previously used to study the relationship between exercise and drug abuse (Wang et al., 2019). These tools facilitated an in-depth analysis of the variables under investigation.

#### Physical exercise rating scale

Several researchers used the physical exercise rating scale (PARS-3) to assess physical activity levels based on three dimensions: intensity, duration, and frequency. Participants rated their activity using a 5-point Likert scale (0 to 4 points). The total exercise score was calculated using the formula: intensity score × (duration score - 1) × frequency score. Scores ranged from 0 to 100, with categories defined as low physical activity (≤19 points), moderate activity (20–42 points), and high activity (≥43 points).

#### Inhibition scale

The inhibition scale measured participants’ inhibitory capacity through 25 items across three dimensions. Participants rated their agreement with each statement using a 5-point Likert scale, with options ranging from “strongly disagree” (1 point) to “strongly agree” (5 points). Higher total scores indicated stronger inhibitory control.

#### Drug craving scale

The drug craving scale evaluates craving intensity across three dimensions using 25 items. Participants rate their agreement on a 5-point Likert scale with options ranging from “no” (1 point) to “very yes” (5 points). Higher scores indicate stronger cravings.

### Cardiorespiratory fitness test

Cardiorespiratory fitness was assessed using the Queens College Step Test (QCST), which indirectly measures maximum oxygen uptake (VO_2max_). This low-intensity, short-duration test is ideal for individuals with weaker physical conditions, such as drug addicts. Participants avoided exercise, caffeine, diet pills, and other substances affecting heart rate the day before testing, wore light clothing, and fasted for 2 h prior. The test began with a three-minute rest to record the resting heart rate. Participants then stepped on a 41.3 cm platform to a metronome beat (88 beats/min for women, 96 beats/min for men) using a four-step cadence (“up-up-down-down”) for 3 min, alternating legs to prevent fatigue. After stopping, the heart rate was measured for 15 s between 3:05 and 3:20, then multiplied by 4 to determine beats per minute (bpm). The testers are online-trained and experienced supervisors for exercise-based drug rehabilitation. VO_2max_ was calculated using the formulas (Mcardle et al., 1972):

Men: $VO2\max\;\left(\mathrm{ml}/\mathrm{kg}/\min\right)=111.33-\left(0.42\;x\;\mathrm{bpm}\right)\text{.}$

Women: $VO2\max\;\left(\mathrm{ml}/\mathrm{kg}/\min\right)=65.81-\left(0.1847\;x\;\mathrm{bpm}\right)\text{.}$

### Research program

After securing biomedical ethical approval, researchers obtained informed consent from leaders, supervisors, medical staff, and drug addicts at the compulsory drug rehabilitation centers. Group tests were conducted in dormitories, with experienced individuals in exercise testing or psychological education serving as main testers. We provided online training to ensure accuracy and consistency in testing procedures. The study used a randomized schedule to administer the scale assessments and the QCST over 2 days. The scale tests were conducted on the first day, followed by QCST the next day. While the were scheduled for the following day.

### Statistical analysis

Data were analyzed using SPSS 21.0 and AMOS 24.0 statistical software packages. Firstly, descriptive statistics, Cronbach’s alpha, exploratory factor analysis, and confirmatory factor analysis were performed to assess scale reliability and validity. Secondly, independent sample t-tests, correlation analysis, regression analysis, and structural equation modeling were applied to examine variable relationships. Besides, the bootstrap method was used to test the mediating effects of cardiorespiratory fitness and inhibition. All statistical significance levels were set at *α* = 0.05.

## Results

### Validity and reliability testing of the inhibition scale and drug craving scale

Exploratory factor analysis of the inhibition scale identified three common factors using the eigenvalue >1 criterion and maximum variance rotation. Nineteen items were retained after removing six due to low identification and contribution rates. The factors were prudence (10 items), self-control (5 items), and self-confidence (4 items), with a cumulative variance contribution rate of 63.37%. Confirmatory factor analysis showed item loadings ranging from 0.63 to 0.89, composite reliability (CR) above 0.70, and average variance extracted (AVE) above 0.50, confirming high validity. Cronbach’s alpha (*α*) coefficients for the overall scale and subdimensions ranged from 0.88 to 0.91, demonstrating strong reliability and validity (Table 2).

**Table 2:** Reliability and validity analysis.

Variables	KMO, Bartlett Sphericity test	Common factors	Items	Cumulative variance (%)	Loading	CR	AVE	Cronbach’s α
Inhibition	KMO = 0.93 *p* < 0.001	Prudence	10	40.17	0.64–0.74	0.91	0.50	0.91
		Self-control	5	55.88	0.68–0.83	0.88	0.60	0.88
		Self-confidence	4	63.37	0.77–0.89	0.90	0.69	0.90
Fit index: *Χ*^2^/df = 1.309; GFI = 0.94; AGFI = 0.92; NFI = 0.94; IFI = 0.99; TLI = 0.98; CFI = 0.99; RMSEA = 0.03								
Drug craving	KMO = 0.94 *p* < 0.001	Drug Cognition	10	44.97	0.65–0.81	0.92	0.54	0.92
		Craving	5	57.02	0.71–0.88	0.91	0.68	0.91
		Irrational belief	5	66.45	0.77–0.85	0.91	0.68	0.91
Fit index: *Χ*^2^/df = 1.56; GFI = 0.92; AGFI = 0.90; NFI = 0.94; IFI = 0.98; TLI = 0.97; CFI = 0.98; RMSEA = 0.04.								

### Validity and reliability testing of the drug craving scale

Exploratory factor analysis of the drug craving scale also extracted three common factors, retaining 20 items after removing five due to low identification and contribution rates. The factors included drug cognition (10 items), drug craving (5 items), and irrational belief (5 items), with a cumulative variance contribution rate of 66.45%. Confirmatory factor analysis revealed item loadings between 0.65 to 0.89, CR above 0.70, and AVE above 0.50, indicating high validity. Cronbach’s α coefficients for the overall scale and subdimensions ranged from 0.91 to 0.92, confirming excellent reliability and validity (Table 2).

### Analysis of influencing factors and correlation

#### Effects of sex on physical exercise, cardiorespiratory fitness, inhibition and drug craving

We used independent sample t-tests to assess how sex affects physical exercise, cardiorespiratory fitness, inhibition, and craving in drug addicts. The results, presented in Table 3, revealed significant sex differences across all variables. Males showed significantly higher levels of physical exercise (*t* = 3.08, *p* < 0.05), cardiorespiratory fitness (*t* = 4.28, *p* < 0.05), and inhibition (*t* = 7.27, *p* < 0.05) compared to females. Conversely, males had significantly lower levels of drug craving than females (*t* = −4.16, *p* < 0.01).

**Table 3:** Effects of sex on physical exercise, cardiorespiratory fitness, inhibition, and drug craving (*n* = 305).

Variables	Sex	*N*	*M*	SD	*T*	*p*
Physical exercise	Male	272	16.28	8.35	3.08	0.003
	Female	33	8.24	3.53		
Cardiorespiratory fitness	Male	272	33.36	5.01	4.28	<0.001
	Female	33	29.39	5.09		
Inhibition	Male	272	66.69	11.58	7.27	<0.001
	Female	33	51.00	12.81		
Drug craving	Male	272	56.36	15.32	−4.16	<0.001
	Female	33	71.18	19.77		

#### Effects of drug types on physical exercise, cardiorespiratory fitness, inhibition and drug craving

We also examined the impact of drug type on physical exercise, cardiorespiratory fitness, inhibition, and drug craving using independent sample t-tests (Table 4). The results showed significant differences between traditional and new drug addicts in inhibition (*t* = 2.50, *p* < 0.05) and drug craving (*t* = −6.93, *p* < 0.01). Traditional drug addictswere significantly higher than that of new drug addicts and exhibited higher inhibition levels and lower drug cravings compared to new drug addicts. However, there were no significant differences between the two groups in terms of physical exercise and cardiorespiratory fitness.

**Table 4:** Effects of drug types on physical exercise, cardiorespiratory fitness, inhibition, and drug craving (*n* = 305).

Variables	Drug Types	*N*	*M*	SD	*T*	*p*
Physical exercise	Traditional drugs	108	17.67	8.16	1.62	0.11
	New drugs	197	14.17	7.91		
Cardiorespiratory fitness	Traditional drugs	108	33.03	5.64	0.24	0.81
	New drugs	197	32.87	4.89		
Inhibition	Traditional drugs	108	67.43	12.51	2.50	0.01
	New drugs	197	63.66	12.60		
Drug craving	Traditional drugs	108	50.01	14.14	−6.93	<0.00
	New drugs	197	62.33	16.07		

#### Effects of years of drug use and physical exercise on cardiorespiratory fitness, inhibition, and drug craving

Table 5 demonstrates a significant negative relationship between the years of drug abuse and physical exercise (*β* = −0.18, *p* < 0.01), cardiorespiratory fitness (*β* = −0.16, *p* < 0.01), and inhibition (*β* = −0.14, *p* < 0.01). In contrast, drug abuse duration was positively related to drug craving (*β* = 0.28, *p* < 0.01). The inclusion of physical exercise variables significantly improved the explanatory power for cardiorespiratory fitness (*R*^2^ = 0.36, *F* = 84.72, *p* < 0.001), inhibition (*R*^2^ = 0.20, *F* = 37.85, *p* < 0.001), and drug craving (*R*^2^ = 0.29, *F* = 62.87, *p* < 0.001). These findings suggest that physical exercise mediates the impact of drug use duration on cardiorespiratory fitness, inhibition, and drug craving.

**Table 5:** Effects of drug years and physical exercise on cardiorespiratory fitness, inhibition, and drug craving.

Variables	Physical exercise	Cardiorespiratory fitness		Inhibition		Drug craving	
	Model 1	Model 2	Model 3	Model 4	Model 5	Model 6	Model 7
Drug years	−0.18	−0.16	−0.06	−0.14	−0.06	0.28	0.19
Physical exercise			0.59		0.43		−0.47
*R* ^2^	0.03	0.03	0.36	0.02	0.200	0.08	0.29
*F*	10.38**	8.33**	84.72***	5.64*	37.854**	5.13**	62.87**

* means *p*-value < 0.01.

#### Correlation between physical exercise, cardiorespiratory fitness, inhibition, and drug craving

Table 6 shows significant positive correlations between physical exercise and cardiorespiratory fitness, as well as with the inhibition components: prudence, self-confidence, and self-control. Furthermore, physical exercise had significant negative correlations with the components of drug craving: drug cognition, craving degree, and irrational belief. Cardiorespiratory fitness also exhibited positive correlations with inhibition components (prudence, self-confidence, and self-control) and negative correlations with drug craving components (drug cognition, craving degree, and irrational belief). Additionally, there were significant negative relationships between the inhibition components and the drug craving components. The correlations of the inhibition or drug craving subscales with the target variables were mirrored in their composite scores.

**Table 6:** Correlation among the physical exercise, cardiorespiratory fitness, inhibition, and drug craving.

Variables	M	SD	1	2	3	4	5	6	7	8
(1) Physical exercise	15.41	8.05	–							
(2) Cardiorespiratory fitness	32.93	5.16	0.60^**^	–						
(3) Prudence	31.43	7.92	0.37^**^	0.24^**^	–					
(4) Self-confidence	18.32	4.83	0.38^**^	0.43^**^	0.43^**^	–				
(5) Self-control	15.24	3.01	0.29^**^	0.32^**^	0.34^**^	0.56^**^	–			
(6) Drug cognition	29.26	8.93	−0.38^**^	−0.38^**^	−0.34^**^	−0.32^**^	−0.20^**^	–		
(7) Craving degree	13.20	5.33	−0.51^**^	−0.54^**^	−0.31^**^	−0.42^**^	−0.39^**^	0.55^**^	–	
(8) Irrational belief	15.51	5.77	−0.40^**^	−0.34^**^	−0.291^**^	−0.27^**^	−0.18^**^	0.47^**^	0.48^**^	–
(9) Inhibition	65.00	12.68	0.44^**^	0.39^**^	0.87^**^	0.79^**^	0.66^**^	−0.38^**^	−0.44^**^	−0.33^**^
(10) Drug craving	57.96	16.48	−0.51^**^	−0.50^**^	−0.38^**^	−0.40^**^	−0.30^**^	0.88^**^	0.79^**^	0.76^**^

**means *p*-value < 0.01.

### Path analysis and model checking

#### Structural model goodness of fit test

**Figure 2:**
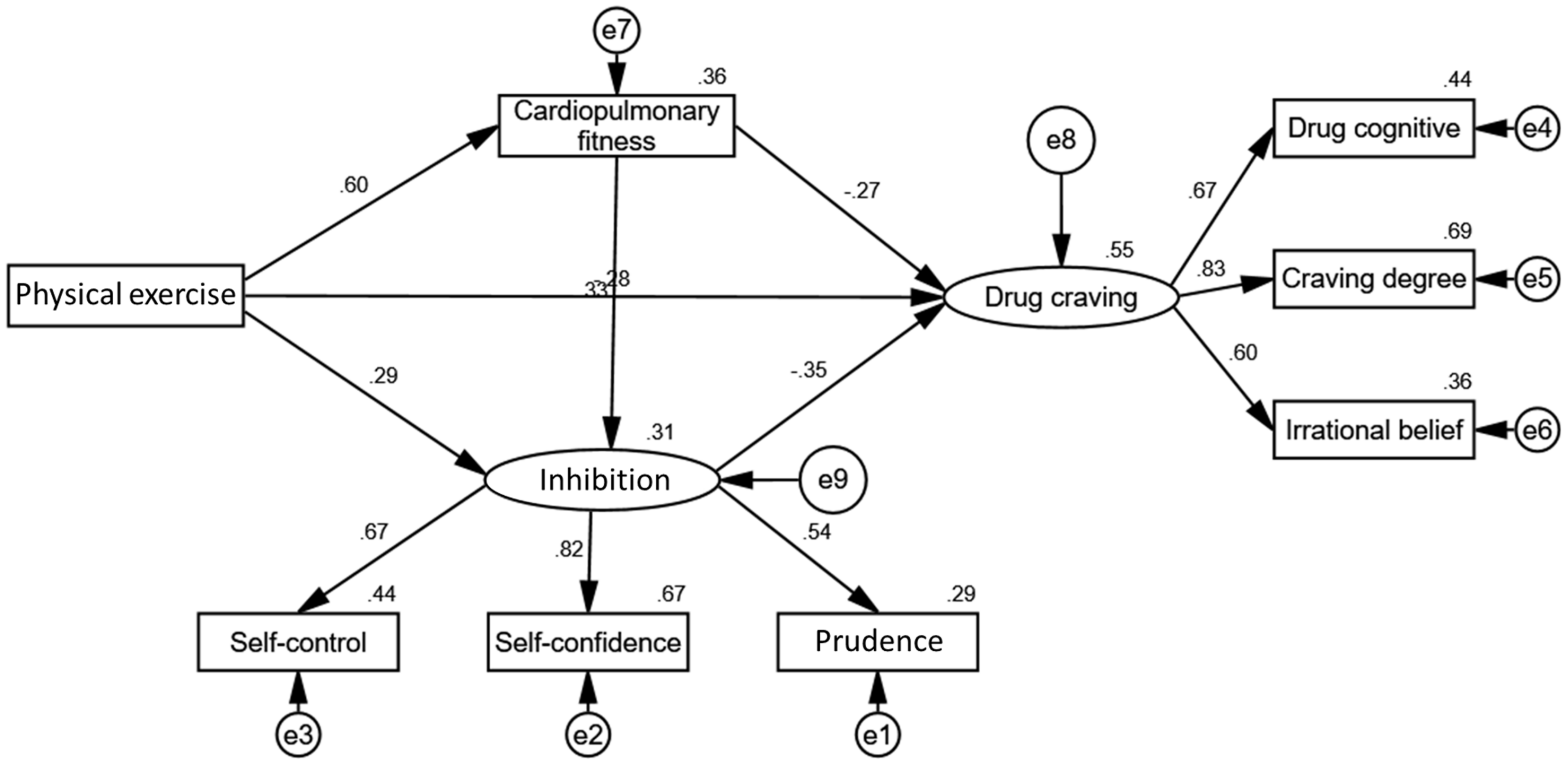
Structural model of physical exercise, cardiorespiratory fitness, inhibition, and drug craving.

AMOS software was used in this study to test the causal relationships between physical exercise, cardiorespiratory fitness, inhibition, and drug craving. After modifications, the structural model diagram was obtained (see Figure 2). The model fit indices were Χ^2^/df = 2.665 (<3), GFI = 0.964, AGFI = 0.919, NFI = 0.947, IFI = 0.966, TLI = 0.940, and CFA = 0.966. All of these values were greater than 0.9, indicating that the model fit the data well.

#### Model test results

Table 7 shows that physical exercise had a significant positive effect on both cardiorespiratory fitness (*β* = 0.60, *p* < 0.01) and inhibition (*β* = 0.29, *p* < 0.01), while significantly reducing drug craving (*β* = −0.28, *p* < 0.01). Greater physical exercise led to better cardiorespiratory fitness and inhibition, and lower drug craving. Cardiorespiratory fitness also had a significant positive effect on inhibition (*β* = 0.33, *p* < 0.01) and a significant negative effect on drug craving (*β* = −0.27, *p* < 0.01). Higher cardiorespiratory fitness resulted in higher inhibition and lower drug craving. Inhibition had a significant negative effect on drug craving (*β* = −0.35, *p* < 0.01), meaning that higher inhibition led to lower drug craving.

**Table 7:** Standardized path coefficient and test result.

Path relationship	*β*	S.E.	*T*	*p*	Result
Physical exercise → Cardiorespiratory fitness	0.60	0.01	12.97	***	Yes
Physical exercise → Inhibition	0.29	0.02	3.74	***	Yes
Cardiorespiratory fitness → Inhibition	0.33	0.07	4.18	***	Yes
Physical exercise→ Drug craving	−0.28	0.02	−4.04	***	Yes
Inhibition→ Drug craving	−0.35	0.12	−4.15	***	Yes
Cardiorespiratory fitness→ Drug craving	−0.27	0.08	−3.94	***	Yes

*** means *p*-value < 0.001.

#### Mediating effect analysis

We used the bootstrap method to examine the mediating effects of cardiorespiratory fitness and inhibition on the relationship between physical exercise and drug craving. By resampling the original data 5,000 times, we calculated the significance of the mediating effect with a 95% confidence interval. Table 8 shows that the indirect effect of physical exercise on drug craving through cardiorespiratory fitness was −0.16 [−0.26, −0.08], indicating that cardiorespiratory fitness significantly mediates the relationship between physical exercise and drug craving. The indirect effect of physical exercise on drug craving through inhibition was −0.10 [−0.29, −0.02], suggesting that inhibition also has a significant mediating role. The chain effect of physical exercise on drug craving through both cardiorespiratory fitness and inhibition was −0.07 [−0.13, −0.03], confirming that cardiorespiratory fitness and inhibition together have a significant chain mediating effect. The three mediating effects accounted for 26.93, 16.42, and 11.17%, respectively.

**Table 8:** Results of mediating effect analysis.

Effect type	Path relationship	Effect size	Bootstrap SE	LLCI	ULCI	Effect ratio
Indirect effect 1	Physical exercise → Cardiorespiratory fitness → Drug craving	−0.16	0.05	−0.26	−0.08	26.93%
Indirect effect 2	Physical exercise → Inhibition → Drug craving	−0.10	0.07	−0.29	−0.02	16.42%
Indirect effect 3	Physical exercise → Cardiorespiratory fitness → Inhibition → Drug craving	−0.07	0.03	−0.13	−0.03	11.17%
Direct effect	Physical exercise → Drug craving	−0.28	0.10	−0.44	−0.06	45.48%
Total indirect effect	Physical exercise → Drug craving	−0.33	0.08	−0.51	−0.22	54.52%
Total effect	Physical exercise → Drug craving	−0.61	0.04	−0.68	−0.53	—

## Discussion and analysis

### Analysis of influencing factors and correlation

#### Sex differences

Our analysis revealed significant gender differences in physical exercise, cardiorespiratory fitness, inhibition, and drug craving. Women comprised a smaller proportion of the survey sample and exhibited lower levels of physical exercise, cardiorespiratory fitness, and inhibition, alongside higher levels of psychological craving compared to men. Previous studies, including our own, have shown that women are more susceptible to drug abuse than men. They often experience stronger initial reactions to drug use, such as euphoria and satisfaction, which increases their risk of addiction. As a result, women also face more intense withdrawal symptoms, mood disorders, and drug cravings, heightening the likelihood of relapse (Zhou et al., 2016; McHugh et al., 2018). These differences may be influenced by fluctuating steroid hormone levels, such as estradiol, progesterone, and testosterone, which contribute to functional and anatomical differences in brain reward circuits between men and women (Maher et al., 2023; Perreault et al., 2023). Furthermore, research confirms that women tend to benefit more from aerobic and mind–body exercises, while men generally prefer anaerobic strength training (Zhou et al., 2016). These findings suggest that sex differences should be a key consideration when designing targeted exercise interventions for drug dependence. Since women exhibit higher psychological cravings, intense withdrawal symptoms, and more emotional disorders, firstly, exercise programs focused on stress and anxiety reduction such as mind–body exercise could be designed (Xu et al., 2024). Secondly, considering women’s deficits in inhibitory functions, exercise interventions aimed at enhancing attention and executive functions should be developed. Additionally, regular assessments and adjustments are recommended during implementation to ensure intervention effectiveness. By tracking and documenting women’s exercise patterns, psychological states, and drug craving levels, exercise plans can be promptly modified to meet individual needs. Our findings emphasize the importance of sex differences in substance dependence and rehabilitation processes. However, current understanding of sex differences still has limitations. For instance, the specific mechanisms through which hormone levels influence drug cravings and recovery remain unclear. Therefore, future research should further investigate the biological basis of sex differences to provide stronger evidence for developing more effective interventions.

#### Drug types

The study revealed that individuals who used traditional drugs had higher inhibition levels and lower cravings compared to those using new drugs. Traditional drugs, extracted and processed from plants, differ significantly from new drugs, which are synthetic stimulants or hallucinogens that directly impact the central nervous system. New drug use is frequently accompanied by symptoms of psychosis, including hyperactivity, heightened alertness, fatigue resistance, and hallucinations. Prolonged abuse of new drugs can lead to brain and neurological diseases, triggering mental and emotional disorders such as paranoia, auditory hallucinations, mania, self-harm, anxiety, and depression (Chavant et al., 2015; Jayanthi et al., 2021). The addiction mechanisms between traditional and new drugs also differ significantly. For example, methamphetamine and similar new drugs act directly on the brain’s central nervous system by promoting the release of neurotransmitters like dopamine (DA), norepinephrine, and serotonin (5-HT), while blocking their reuptake. This results in heightened excitement and continuous stimulation of drug addicts Over time, neurotoxic effects can cause irreversible damage to neurons and structural abnormalities in brain areas like the hippocampus and cingulate gyrus, leading to cognitive issues such as memory and learning difficulties (Jayanthi et al., 2021). The central toxic effects of new drugs are more severe, which aligns with previous research showing that new drug addicts—regardless of the duration of use—tend to have lower inhibitions and higher drug cravings than those using traditional drugs (Wang et al., 2019). This suggests that new drug addicts experience more serious cognitive dysfunction and psychological withdrawal symptoms than traditional drug addicts, mainly due to reduced inhibition, which heightens their drug cravings and increases the likelihood of relapse. Regarding the differences in neural damage between traditional and new drug addicts, exercise interventions require differentiated strategies. The primary goal of exercise interventions for traditional drug should prioritize alleviating physiological dependence and restoring neurological function. For example, low-intensity aerobic exercises (such as walking, yoga) combined with progressive resistance training can be implemented to reduce sympathetic nervous system excitability (Jia et al., 2022). In contrast, the objective for new drug interventions focuses on reshaping reward circuitry and improving executive function. For instance, high-intensity interval training (HIIT) integrated with cognitive tasks can enhance cognitive abilities through dopamine release regulation (Yin et al., 2022). In light of current shortcomings such as the lack of longitudinal tracking studies and unclear neurobiological mechanisms, as well as research priorities like designing differentiated intervention strategies for distinct drug types, future efforts should strengthen mechanism exploration, intervention validation, and translational applications. The ultimate goal is to establish a neuroplasticity-based precision intervention system to reduce emotional disorders, cognitive impairments, and relapse risks during withdrawal from various drugs.

#### Years of drug use

This study found a negative correlation between years of drug use and physical exercise, cardiorespiratory fitness, and inhibition, while it showed a positive correlation with drug craving. The impact of years of drug use on cardiorespiratory fitness, inhibition, and drug craving was amplified by physical exercise. These findings align with previous research, which indicates that the longer someone uses drugs—whether traditional, new, or mixed—the weaker their inhibition and the stronger their craving. Specifically, longer drug use is associated with lower inhibition and higher drug craving (Wang et al., 2019). Additionally, the study observed that individuals who engaged in high levels of physical exercise exhibited higher inhibition and lower drug cravings compared to those with low exercise levels, regardless of their years of drug use (Wang et al., 2019). These results highlight the role of drug use duration in withdrawal and suggest that physical exercise can effectively regulate the impact of long-term drug use on inhibition and cravings. Surprisingly, this study also revealed that as years of drug use increased, physical exercise levels decreased, and cardiorespiratory fitness weakened. These findings suggest that cardiorespiratory fitness may be a crucial factor in how physical exercise influences drug cravings, making it a potential target for addiction treatment. For long-term drug addicts, a gradually progressive exercise intervention strategy is recommended. Initial stages should begin with low-intensity aerobic exercises such as walking or jogging, combined with simple stretching and breathing exercises to improve cardiovascular and respiratory functions. As individuals adapt, exercise intensity and duration can be gradually increased by introducing resistance and strength training to enhance muscle power and endurance. Additionally, combining aerobic and strength training can better restore physical functions and reduce relapse risks (Zarrinkalam et al., 2016). For short-term drug addicts, HIIT is advised. HIIT rapidly enhances cardiovascular capacity, increases energy expenditure, and improves psychological states within short sessions. Through rapid, high-intensity stimulation, it helps break established unhealthy exercise habits and establishes new healthy lifestyle patterns. Furthermore, this study only assessed cardiorespiratory fitness, and future studies should analyze the impact of other components of physical fitness on drug craving.

### Mediating effect analysis

#### The mediating role of inhibition between physical exercise and drug craving

The analysis revealed that physical exercise was negatively correlated with drug craving and positively correlated with inhibition, which in turn was negatively correlated with drug craving and positively correlated with inhibition, which was negatively correlated with drug craving. Inhibition mediated the relationship between physical exercise and drug craving. These results are supported by both animal and human studies, which demonstrate that various forms of physical exercise—such as aerobic exercise, resistance training, tai chi, Qigong, and yoga—can effectively prevent addiction, reduce drug-seeking behaviors, and aid in drug rehabilitation (Zhou et al., 2016; Zhang and Yuan, 2019). Moreover, the study suggests that inhibition plays a key role in how physical exercise helps reduce drug cravings. Research has extensively explored how physical exercise enhances inhibition in drug addicts, and studies indicate an inverted U-shaped relationship between exercise intensity and inhibition (Wang et al., 2016). Likewise, moderate-intensity exercise has been found to be particularly effective in improving inhibition in drug addicts, acting as a mediating factor in reducing cravings (Dongshi et al., 2015; Wang et al., 2016; Wang et al., 2019; Dongshi et al., 2020; Jin et al., 2024). Moderate-intensity exercise acutely enhances neural efficiency through reduced N2 conflict potentials (200–350 ms) and increased theta synchronization (4–7 Hz) in the anterior cingulate cortex, as evidenced by ERP studies (Aly and Kojima, 2020a, Aly and Kojima, 2020b). This neuroelectric priming effect may explain the superiority of moderate-intensity protocols in reducing craving.

Several hypotheses in the literature explain how physical exercise reduces drug dependence, particularly through enhancing inhibition and reducing psychological cravings. These hypotheses include the increase of synaptic plasticity, neurogenesis, catecholamines, endocannabinoids, endogenous opioids, lactic acid, and cardiorespiratory function (Lojovich, 2010; Agbangla et al., 2021; Chen and Nakagawa, 2023). According to drug addiction theory, long-term abuse leads to compensatory changes in the mesencephalic limbic reward system, which involves molecular events in the prefrontal cortex responsible for higher cognitive functions like executive control (Nestler and Lüscher, 2019). Recent neuroelectric evidence extends this understanding, demonstrating that cardiorespiratory fitness modulates prefrontal activity through enhanced P3 amplitudes (450–600 ms) during cognitive control tasks, which revealed that individuals with higher fitness levels show 23% greater P3 amplitudes over frontal-central regions during Go/NoGo tasks, indicating optimized neural resource allocation for inhibition (Aly and Kojima, 2020a, Aly and Kojima, 2020b). These changes disrupt the reward system, impair feedback control, and lead to motivational disorders (Baler and Volkow, 2006). Previous research indicates that exercise can reverse these negative changes by improving synaptic plasticity, neurogenesis, neurotransmitter balance, and transcription factors (Zhou et al., 2016). However, few studies have explored the cardiorespiratory hypothesis to explain how exercise improves inhibition. This gap in research represents an important innovation in our study.

#### Cardiorespiratory fitness as a chain mediator

The study highlighted the crucial role of cardiorespiratory fitness in drug rehabilitation. It found that cardiorespiratory fitness serves as a partial mediator between physical exercise and drug craving. Additionally, cardiorespiratory fitness is positively correlated with inhibition and negatively correlated with psychological craving, suggesting that it is an essential physiological marker for rehabilitation. Cardiorespiratory fitness has been recognized as the fifth vital sign, alongside respiration, body temperature, pulse, and blood pressure (Ross et al., 2016). For healthy individuals, higher cardiorespiratory fitness correlates with better top-down control and improved communication within the brain’s cortex (Hsieh et al., 2020). Besides, cardiorespiratory fitness is closely related to cerebral inhibitory control, and high levels of cardiorespiratory fitness show better and more stable performance on tasks related to inhibitory control (Hsieh et al., 2020). Drug addictss, however, often show impaired cardiorespiratory fitness, which manifests as cardiovascular issues like arrhythmia and sinus bradycardia. These impairments hinder recovery by disrupting sympathetic nerve function and weakening cardiopulmonary fitness (Ciucă Anghel et al., 2023).

As research continues to evolve, the cardiorespiratory hypothesis is increasingly being used to explain how exercise benefits neurocognitive function. High levels of cardiorespiratory fitness enhance cerebral blood flow, metabolic activity, and oxygenation, which in turn improve brain neurotransmitter function and cognition (Dustman et al., 1984; Kramer et al., 1999). In a previous randomized controlled trial, we found that tobacco dependence impaired cardiorespiratory fitness, but both moderate-intensity and high-intensity exercises were effective in improving it (Zhou et al., 2024). Another study showed that 4 weeks of treadmill exercise improved fitness and reduced cravings and cocaine use in individuals with concurrent cocaine and tobacco use disorder (De La Garza 2nd et al., 2016). Our current findings support these results, showing that physical exercise reduces drug cravings by enhancing cardiorespiratory fitness and increasing inhibition. Cardiorespiratory fitness and inhibition serve as chain mediators in the process of exercise intervention for drug cravings. These results suggest that cardiorespiratory fitness should be considered when developing exercise interventions for drug rehabilitation programs. Developmental studies further substantiate this relationship: in preadolescent children, every 1 MET increase in VO2max corresponds to 18.3 ms faster executive attention resolution (Abdelkarim et al., 2023). This implies that fitness-induced neuroplasticity may counteract drug-induced cognitive deficits across the lifespan. Exercise programs that focus on improving cardiorespiratory fitness can significantly benefit the rehabilitation of physical and cognitive functions, while also reducing drug cravings. Thus, our study contributes to the cardiorespiratory hypothesis by further clarifying the mechanisms underlying exercise interventions in drug dependence. However, our study has some limitations, such as only evaluating compulsory inpatients; it is not possible to know whether or how many patients were using psychotropic medications; no mention of whether patients engaged in regular physical exercise, and so on.

## Conclusion

The sex, drug type, and duration of drug use are key factors influencing the effectiveness of exercise interventions in drug rehabilitation. Male drug addicts exhibited better physical exercise capacity, cardiorespiratory fitness, and inhibition compared to females, but their psychological cravings were significantly lower. Additionally, the longer the duration of drug use, the lower the levels of physical exercise, cardiorespiratory fitness, and inhibition, while psychological cravings were higher. The number of years of drug use significantly impacts cardiorespiratory fitness, inhibition, and psychological cravings, particularly through physical exercise.

The mechanism through which physical exercise influences drug cravings is closely linked to cardiorespiratory fitness and inhibition. Physical exercise positively affects cardiorespiratory fitness and inhibition, while negatively impacting psychological cravings. Cardiorespiratory fitness is also positively correlated with inhibition, and both are negatively correlated with cravings. Equally, exercise influences drug cravings through direct pathways and indirect pathways, such as the mediating effects of cardiorespiratory fitness and the chain mediating effect between cardiorespiratory fitness and inhibition.

This study therefore demonstrates that physical exercise plays a crucial role in reducing drug cravings and supporting rehabilitation in drug addicts. It highlights the significant impact of sex, drug type, and the duration of drug use on the effectiveness of exercise interventions. Specifically, male drug addicts show better physical exercise, cardiorespiratory fitness, and inhibition compared to females, while those with longer drug use histories experience lower physical exercise levels and greater cravings. Furthermore, the findings also emphasize that improvements in cardiorespiratory fitness and inhibition through physical exercise serve as key mechanisms for reducing psychological cravings. Overall, this study underscores the importance of integrating physical exercise, particularly those aimed at enhancing cardiorespiratory fitness, in drug rehabilitation programs to improve both physical and cognitive function while reducing drug cravings.

Based on the findings, it is therefore recommended that drug rehabilitation programs incorporate tailored physical exercise routines to enhance cardiorespiratory fitness and inhibition, which can help reduce drug cravings and improve overall rehabilitation outcomes. Additionally, according to the rehabilitation patients’ drug use history (sex, drug types, years of drug use), interventions can selectively incorporate aerobic exercises, mind–body exercises, and resistance training of varying intensities either individually or in combination. However, this study has some limitations, including the reliance on self-reported data and the lack of long-term follow-up to assess the sustainability of the exercise intervention’s effects. Future research should explore the long-term impact of exercise interventions on drug craving reduction, consider different types of exercise modalities, and examine their effects across diverse populations, including those with varying levels of drug dependence. Additionally, studies should investigate the specific physiological mechanisms, such as the role of neurotransmitters, in the relationship between exercise, cardiorespiratory fitness, and drug addiction.

## Data Availability

The original contributions presented in the study are included in the article/supplementary material, further inquiries can be directed to the corresponding author.

## References

[ref1] Abdelkarim O. Aly M. ElGyar N. Shalaby A. M. Kamijo K. Woll A.. (2023). Association between aerobic fitness and attentional functions in Egyptian preadolescent children. Front. Psychol. 14:1172423. doi: 10.3389/fpsyg.2023.1172423, PMID: 37484080 PMC10359903

[ref2] Agbangla N. F. Maillot P. Vitiello D. (2021). Mini-review of studies testing the cardiorespiratory hypothesis with near-infrared spectroscopy (NIRS): overview and perspectives. Front. Neurosci. 15:699948. doi: 10.3389/fnins.2021.699948 34456672 PMC8387658

[ref3] Aly M. Kojima H. (2020a). Acute moderate-intensity exercise generally enhances neural resources related to perceptual and cognitive processes: a randomized controlled ERP study. Ment. Health Phys. Act. 19:100363. doi: 10.1016/j.mhpa.2020.100363

[ref4] Aly M. Kojima H. (2020b). Relationship of regular physical activity with neuroelectric indices of interference processing in young adults. Psychophysiology 57:e13674. doi: 10.1111/psyp.13674, PMID: 33460156

[ref5] Baler R. D. Volkow N. D. (2006). Drug addiction: the neurobiology of disrupted self-control. Trends Mol. Med. 12, 559–566. doi: 10.1016/j.molmed.2006.10.005, PMID: 17070107

[ref6] Billinger S. A. Vidoni E. D. Morris J. K. Thyfault J. P. Burns J. M. (2017). Exercise test performance reveals evidence of the cardiorespiratory fitness hypothesis. J. Aging Phys. Act. 25, 240–246. doi: 10.1123/japa.2015-0321, PMID: 27705069 PMC5374040

[ref7] Brown R. A. Abrantes A. M. Read J. P. Marcus B. H. Jakicic J. Strong D. R.. (2010). A pilot study of aerobic exercise as an adjunctive treatment for drug dependence. Ment. Health Phys. Act. 3, 27–34. doi: 10.1016/j.mhpa.2010.03.001, PMID: 20582151 PMC2889694

[ref8] Chavant F. Boucher A. Le Boisselier R. Deheul S. Debruyne D. (2015). New synthetic drugs in addictovigilance. Therapie 70, 167–189. doi: 10.2515/therapie/2015001, PMID: 25858573

[ref9] Chen C. Nakagawa S. (2023). Physical activity for cognitive health promotion: an overview of the underlying neurobiological mechanisms. Ageing Res. Rev. 86:101868. doi: 10.1016/j.arr.2023.101868, PMID: 36736379

[ref10] Ciucă Anghel D. M. Nițescu G. V. Tiron A. T. Guțu C. M. Baconi D. L. (2023). Understanding the mechanisms of action and effects of drugs of abuse. Molecules 28:4969. doi: 10.3390/molecules28134969, PMID: 37446631 PMC10343642

[ref11] Committee, C. n. n. c. (2024). "China drug situation Report" Available online at:http://www.nncc626.com/2021xb/gjjdb/index.htm (Accessed 19, June 2024).

[ref12] De La Garza R. 2nd Yoon J. H. Thompson-Lake D. G. Haile C. N. Eisenhofer J. D. Newton T. F.. (2016). Treadmill exercise improves fitness and reduces craving and use of cocaine in individuals with concurrent cocaine and tobacco-use disorder. Psychiatry Res. 245, 133–140. doi: 10.1016/j.psychres.2016.08.003, PMID: 27541349 PMC5067203

[ref13] Ding F. Jia S. Wang P. Liu C. Li Y. (2024). Effect of exercise on cravings levels in individuals with drug dependency: a systematic review. Addict. Behav. 158:108127. doi: 10.1016/j.addbeh.2024.108127 39127026

[ref14] Dongshi W. Chenglin Z. Chang Y. K. (2015). Acute exercise ameliorates craving and inhibitory deficits in methamphetamine: an ERP study. Physiol. Behav. 147, 38–46. doi: 10.1016/j.physbeh.2015.04.008 25846839

[ref15] Dongshi W. Zhu T. Chen J. Lu Y. Chang Y. K. (2020). Acute aerobic exercise ameliorates cravings and inhibitory control in heroin addicts: evidence from event-related potentials and frequency bands. Front. Psychol. 11:561590. doi: 10.3389/fpsyg.2020.561590 33101132 PMC7554636

[ref16] Dustman R. E. Ruhling R. O. Russell E. M. Shearer D. E. Bonekat H. W. Shigeoka J. W.. (1984). Aerobic exercise training and improved neuropsychological function of older individuals. Neurobiol. Aging 5, 35–42. doi: 10.1016/0197-4580(84)90083-6, PMID: 6738784

[ref17] Fischer J. Butt C. Dawes H. Foster C. Neale J. Plugge E.. (2012). Fitness levels and physical activity among class a drug users entering prison. Br. J. Sports Med. 46, 1142–1144. doi: 10.1136/bjsports-2011-090724, PMID: 22522587

[ref18] Garber C. E. Blissmer B. Deschenes M. R. Franklin B. A. Lamonte M. J. Lee I. M.. (2011). American College of Sports Medicine position stand. Quantity and quality of exercise for developing and maintaining cardiorespiratory, musculoskeletal, and neuromotor fitness in apparently healthy adults: guidance for prescribing exercise. Med. Sci. Sports Exerc. 43, 1334–1359. doi: 10.1249/MSS.0b013e318213fefb, PMID: 21694556

[ref19] Goldstein R. Z. Volkow N. D. (2011). Dysfunction of the prefrontal cortex in addiction: neuroimaging findings and clinical implications. Nat. Rev. Neurosci. 12, 652–669. doi: 10.1038/nrn3119, PMID: 22011681 PMC3462342

[ref20] Hsieh S. S. Chueh T. Y. Morris T. P. Kao S. C. Westfall D. R. Raine L. B.. (2020). Greater childhood cardiorespiratory fitness is associated with better top-down cognitive control: a midfrontal theta oscillation study. Psychophysiology 57:e13678. doi: 10.1111/psyp.13678, PMID: 32877574

[ref21] Jayanthi S. Daiwile A. P. Cadet J. L. (2021). Neurotoxicity of methamphetamine: Main effects and mechanisms. Exp. Neurol. 344:113795. doi: 10.1016/j.expneurol.2021.113795 34186102 PMC8338805

[ref22] Jia D. Zhou J. Xu Y. (2022). Effectiveness of traditional Chinese health-promoting exercise as an adjunct therapy for drug use disorders: a systematic review and Meta-analysis. J. Integr. Complement. Med. 28, 294–308. doi: 10.1089/jicm.2021.0285, PMID: 35426734

[ref23] Jin J. Zhai X. Taylor A. Zhu T. Wang D. Peng B.. (2024). Dose–response effects of resistance exercise on ameliorating cravings and executive functions in individuals with methamphetamine use disorders. Ment. Health Phys. Act. 27:100633. doi: 10.1016/j.mhpa.2024.100633

[ref24] Kramer A. F. Hahn S. Cohen N. J. Banich M. T. McAuley E. Harrison C. R.. (1999). Ageing, fitness and neurocognitive function. Nature 400, 418–419, PMID: 10440369 10.1038/22682

[ref25] Lojovich J. M. (2010). The relationship between aerobic exercise and cognition: is movement medicinal? J. Head Trauma Rehabil. 25, 184–192. doi: 10.1097/HTR.0b013e3181dc78cd, PMID: 20473092

[ref26] Lynch W. J. Peterson A. B. Sanchez V. Abel J. Smith M. A. (2013). Exercise as a novel treatment for drug addiction: a neurobiological and stage-dependent hypothesis. Neurosci. Biobehav. Rev. 37, 1622–1644. doi: 10.1016/j.neubiorev.2013.06.011, PMID: 23806439 PMC3788047

[ref27] Maher E. E. Strzelecki A. M. Weafer J. J. Gipson C. D. (2023). The importance of translationally evaluating steroid hormone contributions to substance use. Front. Neuroendocrinol. 69:101059. doi: 10.1016/j.yfrne.2023.101059 36758769 PMC10182261

[ref28] Mcardle W. D. Katch F. I. Pechar G. S. Jacobson L. Ruck S. (1972). Reliability and interrelationships between maximal oxygen intake, physical work capacity and step-test scores in college women. Med. Sci. Sports 4, 182–186. doi: 10.1249/00005768-197200440-00019, PMID: 4648576

[ref29] McHugh R. K. Votaw V. R. Sugarman D. E. Greenfield S. F. (2018). Sex and gender differences in substance use disorders. Clin. Psychol. Rev. 66, 12–23. doi: 10.1016/j.cpr.2017.10.012, PMID: 29174306 PMC5945349

[ref30] Morris L. Stander J. Ebrahim W. Eksteen S. Meaden O. A. Ras A.. (2018). Effect of exercise versus cognitive behavioural therapy or no intervention on anxiety, depression, fitness and quality of life in adults with previous methamphetamine dependency: a systematic review. Addict. Sci. Clin. Pract. 13:4. doi: 10.1186/s13722-018-0106-4, PMID: 29338767 PMC5771022

[ref31] Nations. (2024). "World Drug Report." Available online at:https://www.unodc.org/unodc/en/data-and-analysis/world-drug-report-2024.html (Accessed June 26, 2024).

[ref32] Nestler E. J. Lüscher C. (2019). The molecular basis of drug addiction: linking epigenetic to synaptic and circuit mechanisms. Neuron 102, 48–59. doi: 10.1016/j.neuron.2019.01.016 30946825 PMC6587180

[ref33] P.R.C, M. O. J. (2024). "Government information publicity." Available online at: https://www.moj.gov.cn/pub/sfbgw/zwxxgk/fdzdgknr/fdzdgknrjdhy/202406/t20240626_501171.html (Accessed 26 June, 2024).

[ref34] Perreault B. Hammond N. Thanos P. K. (2023). Effects of exercise on testosterone and implications of drug abuse: a review. Clin. Neuropharmacol. 46, 112–122. doi: 10.1097/WNF.0000000000000546, PMID: 37191565

[ref35] Ross R. Blair S. N. Arena R. Church T. S. Després J. P. Franklin B. A.. (2016). Importance of assessing cardiorespiratory fitness in clinical practice: a case for fitness as a clinical vital sign: a scientific statement from the American Heart Association. Circulation 134, e653–e699. doi: 10.1161/CIR.0000000000000461, PMID: 27881567

[ref36] Wang X. Y. Li Y. Li J. Hao W. (2023). Emerging patterns of substance abuse and related treatment in China. Curr. Opin. Psychiatry 36, 277–282. doi: 10.1097/YCO.0000000000000878, PMID: 37191649

[ref37] Wang K. Luo J. Zhang T. Ouyang Y. Lu Y. (2019). Effect of physical activity on drug craving of women with substance use disorder in compulsory isolation: mediating effect of internal inhibition. Front. Psychol. 10:1928. doi: 10.3389/fpsyg.2019.01928, PMID: 31551851 PMC6733992

[ref38] Wang D. Zhou C. Zhao M. Wu X. Chang Y. K. (2016). Dose–response relationships between exercise intensity, cravings, and inhibitory control in methamphetamine dependence: an ERPs study. Drug Alcohol Depend. 161, 331–339. doi: 10.1016/j.drugalcdep.2016.02.023, PMID: 26946990

[ref39] Weber R. J. Gomez-Flores R. Smith J. E. Martin T. J. (2004). Immune, neuroendocrine, and somatic alterations in animal models of human heroin abuse. J. Neuroimmunol. 147, 134–137. doi: 10.1016/j.jneuroim.2003.10.029, PMID: 14741445

[ref40] Weinstock J. Farney M. R. Elrod N. M. Henderson C. E. Weiss E. P. (2016). Exercise as an adjunctive treatment for substance use disorders: rationale and intervention description. J. Subst. Abus. Treat. 72:40–47. doi: 10.1016/j.jsat.2016.09.002 PMC5289308 27666958

[ref41] Xu H. Liu J. Li P. Liang Y. (2024). Effects of mind-body exercise on perimenopausal and postmenopausal women: a systematic review and meta-analysis. Menopause 31, 457–467. doi: 10.1097/GME.0000000000002336, PMID: 38669625 PMC11465887

[ref42] Yin Y. Yang S. Xiao K. Wang T. Wang J. Schöllhorn W. I.. (2022). Comparison of the acute effects of tai chi versus high-intensity interval training on inhibitory control in individuals with substance use disorder. Front. Psychol. 13:941719. doi: 10.3389/fpsyg.2022.941719 36267065 PMC9577467

[ref43] Zarrinkalam E. Heidarianpour A. Salehi I. Ranjbar K. Komaki A. (2016). Effects of endurance, resistance, and concurrent exercise on learning and memory after morphine withdrawal in rats. Life Sci. 157, 19–24. doi: 10.1016/j.lfs.2016.05.034, PMID: 27234896

[ref44] Zhang M. Jia J. Yang Y. Zhang L. Wang X. (2023). Effects of exercise interventions on cognitive functions in healthy populations: a systematic review and meta-analysis. Ageing Res. Rev. 92:102116. doi: 10.1016/j.arr.2023.102116 37924980

[ref45] Zhang T. Wang K. Li N. Hurr C. Luo J. (2021). The relationship between different amounts of physical exercise, internal inhibition, and drug craving in individuals with substance-use disorders. IJERPH 18:12436. doi: 10.3390/ijerph182312436 34886162 PMC8656815

[ref46] Zhang T. Wang K. Qu M. Jiang H. Chen X. Luo J. (2020). The effect of physical activity on drug cravings of drug addicts with AIDS: the dual mediating effect of internal inhibition. Front. Psychol. 11:2002. doi: 10.3389/fpsyg.2020.02002 33117203 PMC7566164

[ref47] Zhang L. Yuan T. F. (2019). Exercise and substance abuse. Int. Rev. Neurobiol. 147, 269–280. doi: 10.1016/bs.irn.2019.07.007, PMID: 31607357

[ref48] Zhou Y. Feng W. Zhang N. Guo J. Xu S. Wang S.. (2024). Effects of different exercise interventions on cardiopulmonary function in male tobacco-dependent college students. J. Sports Sci. 42, 1323–1330. doi: 10.1080/02640414.2024.2390303, PMID: 39133775

[ref49] Zhou Y. Zhao M. Zhou C. Li R. (2016). Sex differences in drug addiction and response to exercise intervention: from human to animal studies. Front. Neuroendocrinol. 40, 24–41. doi: 10.1016/j.yfrne.2015.07.001, PMID: 26182835 PMC4712120

